# Calibration Assessment of Low-Cost Carbon Dioxide Sensors Using the Extremely Randomized Trees Algorithm

**DOI:** 10.3390/s23136153

**Published:** 2023-07-04

**Authors:** Tiago Araújo, Lígia Silva, Ana Aguiar, Adriano Moreira

**Affiliations:** 1 Federal Institute of Education, Science and Technology of Rio Grande do Norte (IFRN), Parnamirim 59124-455, Brazil; tiago.araujo@ifrn.edu.br; 2 Algoritmi Research Centre, University of Minho, 4800-058 Guimarães, Portugal; 3 CTAC Research Centre, University of Minho, 4800-058 Guimarães, Portugal; lsilva@civil.uminho.pt; 4 Telecommunications Institute, Engineering Faculty, University of Porto, 4200-465 Porto, Portugal; anaa@fe.up.pt

**Keywords:** environmental monitoring, carbon dioxide sensors, machine learning, sensor calibration

## Abstract

As the monitoring of carbon dioxide is an important proxy to estimate the air quality of indoor and outdoor environments, it is essential to obtain trustful data from CO_2_ sensors. However, the use of widely available low-cost sensors may imply lower data quality, especially regarding accuracy. This paper proposes a new approach for enhancing the accuracy of low-cost CO_2_ sensors using an extremely randomized trees algorithm. It also reports the results obtained from experimental data collected from sensors that were exposed to both indoor and outdoor environments. The indoor experimental set was composed of two metal oxide semiconductors (MOS) and two non-dispersive infrared (NDIR) sensors next to a reference sensor for carbon dioxide and independent sensors for air temperature and relative humidity. The outdoor experimental exposure analysis was performed using a third-party dataset which fit into our goals: the work consisted of fourteen stations using low-cost NDIR sensors geographically spread around reference stations. One calibration model was trained for each sensor unit separately, and, in the indoor experiment, it managed to reduce the mean absolute error (MAE) of NDIR sensors by up to 90%, reach very good linearity with MOS sensors in the indoor experiment (r^2^ value of 0.994), and reduce the MAE by up to 98% in the outdoor dataset. We have found in the outdoor dataset analysis that the exposure time of the sensor itself may be considered by the algorithm to achieve better accuracy. We also observed that even a relatively small amount of data may provide enough information to perform a useful calibration if they contain enough data variety. We conclude that the proper use of machine learning algorithms on sensor readings can be very effective to obtain higher data quality from low-cost gas sensors either indoors or outdoors, regardless of the sensor technology.

## 1. Introduction

Widely available, affordable sensors are gaining the attention of specialized and unspecialized individuals due to their promise of reaching an acceptable sense of reality at significantly lower costs when compared to professional commercial solutions. The use of low-cost sensors is being considered not only in relatively newer areas, such as participatory sensing [1,2] and smart cities [3,4,5], but also in consolidated fields of study, such as environmental monitoring itself [6,7], always on the ground that the reduced cost of these devices helps to increase the density of a data collection mesh and, consequently, to expand the probability of achieving higher quality results due to the richer spatial granularity of the sensing system [8].

Considering a global context where there is an increase in environmental awareness, especially regarding air pollution, efforts to use low-cost sensors to monitor air quality have also found fertile ground [9]. Since the environment directly affects the quality of life and health of individuals, including breathable air, it is desirable to have a proper community air quality monitoring system as well as an appropriate assessment model [10]. Several projects have been developed in this context recently. For example, the authors in [11] proposed a wireless low-powered device using low-cost gas sensors to monitor the air quality in smart cities and smart home contexts. Hu et al. [12] considered the mobility of vehicles in urban spaces and proposed an air-quality system with low-cost sensors to be applied in public vehicles to crowd-source data about air pollution in the city and convert it into real-time maps. The authors in [13] report a study using low-cost sensors for different pollutants in an urban space aiming to identify regions with a prevalence of respiratory diseases. A comprehensive review of low-cost air quality sensor applications is reported by Karagulian et al. [14].

Yet, low-cost sensors may present a tacit trade-off regarding their data quality and, sometimes, the accurate calibration of such sensors may become a resource- and time-consuming process. Given this, studies addressing sensor performance and data quality enhancement alternatives have become necessary, as in the process described by Russel et al. [15], where they present a hybrid calibration process. Jiao et al. [16] assessed the performance of low-cost sensors for gases and observed the need for data quality enhancement since mixed results from the sensors were observed. Similar conclusions were presented by Munir et al. [17], in a case study with field calibration of low-cost sensors, in which part of the evaluated sensors presented very good results. Gaynullin et al. presented an advanced algorithm to compensate for the interference of atmospheric pressure variations when dealing with non-dispersive infrared sensors for environmental measurements [18]. Within the citizen science approach, the authors in [19] reported the outcomes of an experiment with low-cost commercially available sensors for ozone and nitrogen dioxide deployed into the field using nearby professional stations as references for the measurements and found good results for the ozone sensors but no consistent results for the nitrogen dioxide sensors. Although linear regression is, perhaps, the most intuitive resource in enhancing the accuracy of sensors in general, sometimes the dependence between sensor data and the reference might not be linear or unidimensional, as observed by Moreno-Rangel et al. [20]. In such nonlinear-multidimensional sensor dependency scenarios, the use of machine learning (ML) algorithms can be a useful tool in the calibration process [21,22,23]. Thus, in this current context of distributed, participatory, and decentralized sensing, data quality becomes an even more relevant concern because it is fundamental to achieving meaningful results [24].

Low-cost widely available sensors, provided that they present satisfactory accuracy, can also play an important role in indoor air quality assessment. Although carbon dioxide is not considered a toxic gas, in higher concentrations, it has some side effects in humans. In the outdoor environment, CO_2_ concentration levels are globally assessed due to their relationship with the greenhouse effect, wildfires, and fuel burning. When indoors, the approach is different because the main sources of carbon dioxide in this environment are the occupants, and occupied rooms without proper ventilation tend to accumulate CO_2_ over time. Considering that the quantity of CO_2_ in each indoor space can be proportional to the number of occupants, studies are using gas concentration levels to estimate room occupancy [25,26,27]. Moreover, CO_2_ levels are a good indicator of air quality and ventilation system performance. The main concern is that higher carbon dioxide concentration levels may cause undesirable effects in occupants, which can include dizziness, lack of initiative, headache, and other cognitive impairments [28,29,30,31]. There are even more worrying scenarios, for example, in primary schools with poor (or inexistent) ventilation systems. Increased concentration levels of carbon dioxide in classrooms may affect children’s concentration and learning [32].

Therefore, whether as a situation diagnosis or as a continuous observation tool, the monitoring of carbon dioxide indoors is relevant mainly because of the impacts that the gas can cause on the performance and productivity of workers or students when in higher concentrations. High-accuracy measuring instruments serve this purpose satisfactorily but are expensive. This impedes their acquisition, especially in developing countries, public schools, or large facilities. This creates an opportunity to apply low-cost carbon dioxide sensors, even though further treatment may be required to improve the accuracy of their readings. This is especially so for metal oxide semiconductor sensors (MOS), which are dependent on environmental conditions [33] and whose information extraction process from the nominal response curve provided by the manufacturer is non-trivial or subject to large inaccuracy [34].

In this context, this article reports on the results of a new approach to the accuracy enhancement of low-cost carbon dioxide sensors using an extremely randomized trees algorithm [35]. Each sensor unit has an individual calibration model, which was trained with a dataset composed of the aggregation of the carbon dioxide readings from the sensor itself and environmental readings from low-cost temperature and humidity sensors. All data were obtained from experimental exposure to real-world, uncontrolled scenarios. The target data were obtained from a high-accuracy instrument’s readings. In the indoor environment experiment, we conducted an experiment where the sensors were exposed to different situations of room occupancy. This provided a wide range of carbon dioxide concentration levels over time, to assess the accuracy that can be obtained with these devices through the presented calibration approach, in a broad range of values. Regarding the outdoor scenario, we used a dataset from an experiment reported by Zimmermann et al. [22], in which they built fourteen stations using low-cost NDIR sensors and placed them in the geographic vicinity of reference stations.

The next sections describe the materials and methods, results and data analysis, the discussion, and, lastly, the conclusions.

## 2. Materials and Methods

The dataset used in the indoor experiment was obtained from a set of low-cost sensor readings that were placed amongst a reference instrument in rooms with mixed occupations over time, creating different scenarios. The next subsections describe which sensors were used, the experimental design, the use of the third-party dataset, details regarding the machine-learning process used, and the performance metrics adopted.

### 2.1. Indoors Experiment

**Figure 1:**
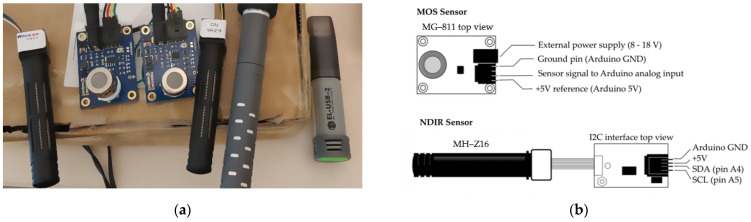
Sensors used in this work. (**a**) From left to right: MH–Z16 (1); 2 x MG–811; MH–Z16 (2); Vaisala GM70 probe; Lascar Electronics sensor. (**b**) Schematic diagram to connect the sensors to the Arduino Uno board.

Among the available commercial off-the-shelf sensors for carbon dioxide monitoring, two low-cost sensor models from two different technologies were chosen, based on their price: MG-811, a metal oxide semiconductor (MOS) sensor that costs around USD 50 [36], and the MH-Z16, a non-dispersive infrared (NDIR) sensor that costs around USD 60 [37]. Two units of each were used. The sensors were wired into two Arduino Uno devices for control and data logging (one sensor model per microcontroller). Also, one Vaisala©GM70 [38], a fully calibrated commercial monitor (±2% of reading + 1.5% of the range), was used as a reference for CO_2_, and one Lascar Electronics EL-2 datalogger [39] was used as a temperature and relative humidity provider. Figure 1 illustrates an arrangement of the sensors used in this paper as well as a simplified diagram of their electronic connection to the Arduino Uno Board.

All devices had their sampling rates set to one sample per minute and were timely synchronized before the experiments. Regarding the Vaisala instrument, its internal memory allows it to save only up to 2700 samples. With the given sample rate, it resulted in a maximum continuous observation period of 45 h. The maximum clock drift observed was one sample for every 7.5 h of the experiment.

**Figure 2:**
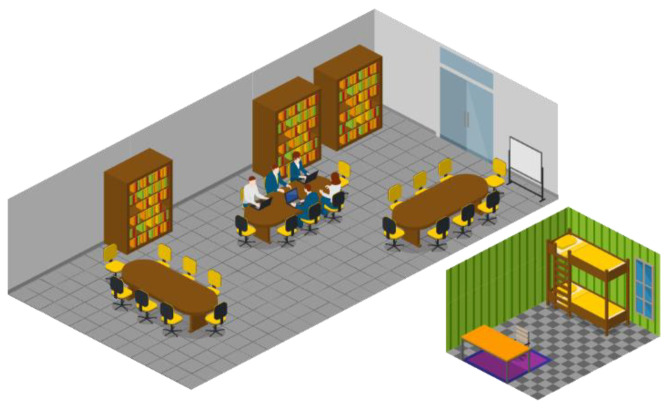
Approximate layout of the indoor environments where the sensors were exposed to carbon dioxide concentrations produced in the room. Office room on the left and the bedroom on the right.

The experiment is based on the exposure of the sensors to indoor environments where the air is expected to have random and non-steady concentrations of carbon dioxide over time. The places chosen for sensor placement were an office room and a dormitory bedroom (Figure 2). The office room has an active ventilation system, measures about 235 m^3^ (12 × 6.5 × 3 m), and is a shared place for researchers in a university, so its occupation varies during the daytime. The bedroom has no active ventilation system (only windows), measures about 30 m^3^ (4 × 3 × 2.5 m), and is occupied by two people during night-time.

In all measurements, the sensors were positioned as close as possible to the centre of the room, with no relevant spacing between them. They were also at least one meter away from a fixed occupant to avoid interference from direct exposure to the breath stream (a stimulus that might not be detected by all sensors).

The sequence of events was designed to attempt to cover a wide variety of situations. Three exposure executions were performed, each one lasting 45 h (the reference instrument data-log limit). The first and third measurements happened in the office room. The second experimental exposure happened in the bedroom, which had the windows closed on the first night, and opened on the second. A hiatus of at least one week was considered between each measurement execution.

The raw dataset was created with data from each unit separately, resulting in 7 dimensions: NDIR #1 and #2, MOS #1 and #2, temperature, humidity, and CO_2_ reference. Each subsequent experimental dataset was joined to the previous one, respecting the data segmentation according to the sensor unit. It resulted in a final length of 135 h (or 8100 samples per sensor unit).

### 2.2. Outdoors Experiment

An outdoor environment is more challenging. Outdoors, these devices are exposed to more unfavourable conditions due to significant variations in temperature and relative humidity, for example. CO_2_ concentrations are also lower. Thus, it is also desirable to test the proposed approach on the calibration of the sensors in an outdoor environment.

Considering that a well-designed mid or long-term outdoor experiment would require longer time and more resources to build and maintain the sensing nodes, we opted to use an open-access dataset resulting from an experiment that fits our objectives.

After reviewing recent papers on this subject, we found the dataset provided by Zimmermann et al. [22], stored at the open-access repository Zenodo. For the experiment, they built fourteen stations for air quality monitoring, containing several sensors for air pollutants. One of these sensing units is the SST CO_2_S-A, a carbon dioxide NDIR sensor that also provides temperature and relative humidity readings. The reference instrument was the CO_2_ analyser LI-820, from LI-COR^®^. The field study took place in Pittsburgh, USA, between August 2016 and February 2017. The sensing platforms were synchronized to provide a reading every 15 min and were positioned around reference stations which were recalibrated periodically. We have observed intermittency on all carbon dioxide sensor data, which means that none of the CO_2_ sensing units worked continuously during all the experiment timespans.

In their paper [22], they used the random forest algorithm trained using all the available data from each platform (including other pollutants sensor output). Our work differs in several points, where we can cite: the algorithm used (the ExtRa-Trees is slightly less computationally costly); the size of the dataset used for training; the split process of training and testing subsets; the features used in the training process, where we included the exposition time of the sensor as a feature in the algorithm.

### 2.3. Data Analysis and Performance Metrics

**Figure 3:**
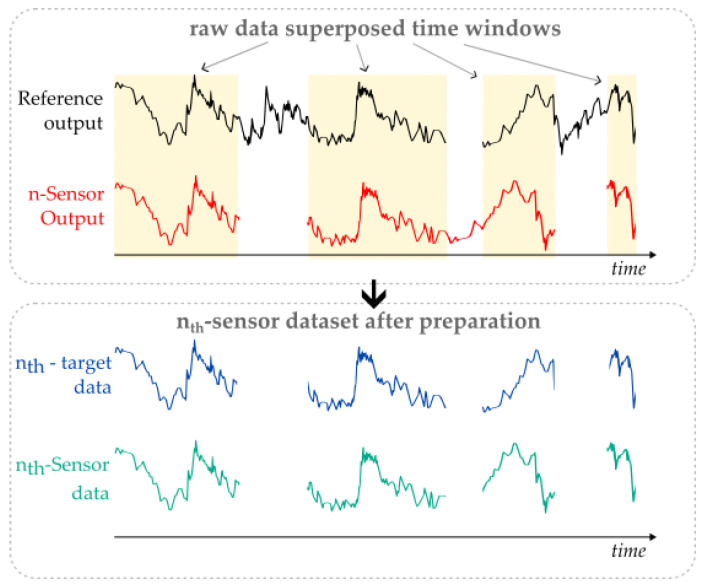
Graphic exemplification of how each sensor dataset was prepared.

**Figure 4:**
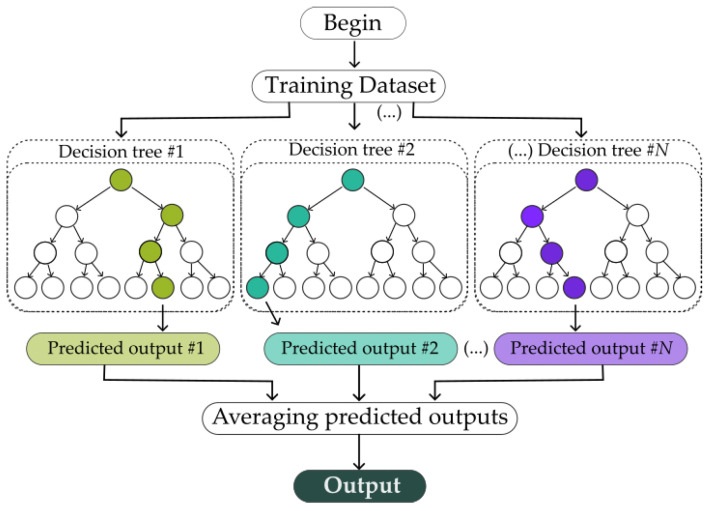
Simplified diagram of the ExtRa-Trees algorithm regression process.

The first step of the data analysis is to prepare the sensor datasets to be used in the algorithm through removing any non-numerical entry and non-synchronous data between each sensor and the reference to ensure that each data entry will have a valid input with a respective output (Figure 3). This process was performed using Python-language-built routines. To determine the most suitable algorithm for our specific scenario, we performed a preliminary benchmark test to compare the performance of different machine learning regressors: the random forests, the extremely randomized trees and Gradient Boosting from the SciKit Learn package available for Python [40]. As we found no background literature that appoints any specific algorithm as the best solution for this kind of application, we opted for the algorithm with the higher accuracy on our tests over the practical data, which was the extremely randomized Trees algorithm (“ExtRa-Trees”). The ExtRa-Trees algorithm is an ensemble learning method that combines the concept of random decision forests with additional randomness in the selection of split points. Like random forests, ExtRa-Trees builds multiple decision trees using samples of the training data. However, what sets ExtRa-Trees apart is that it does use the whole training data instead of bootstrapping it and, also, for each tree and at each split node, it selects the splitting feature randomly from a subset of features, and the split point is chosen randomly instead of being optimized. This additional level of randomness helps to reduce overfitting and enhance the diversity of the ensemble. A simplified diagram of how ExtRa-Trees works is shown in Figure 4. Ultimately, ExtRa-Trees produces a robust and accurate regression model with relatively low computational cost [35].

As ExtRa-Trees is a supervised machine learning model, it needs two data categories to perform the modelling—the input (either unidimensional or multidimensional) and the target (usually unidimensional). Thus, the sensor readings need to be separated into these groups. The target data were composed of the readings obtained from the reference instrument in parts per million (ppm). The input dataset, in turn, was formed separately for each sensor unit containing readings from carbon dioxide (from the sensor that will be calibrated), temperature, and relative humidity (measured in place), and in the outdoor analysis, the running time of the evaluated sensor in the form of day fraction.

To build the training dataset, we used a k-fold for splitting and shuffling with the initial value of k equal to 5 and picked the smaller part (20%) as the training subset. In each execution of the split and shuffle process (k-fold), the training subset was obtained through randomly picking new points throughout the original dataset. The final performance was then obtained through averaging these execution outputs. It is important to emphasize that the application of the ExtRa-Trees algorithm to time measurements resembles the sub-ensemble approach used in stochastic modelling. In stochastic modelling, it is mandatory to maintain the statistical representation of the process within the sub-ensembles. In our work, we did this through building the training dataset through random sampling throughout the sensor dataset, so the statistical parameters that may be different over time could be maintained in the training dataset. To investigate the performance of the algorithm when trained with different information quantities, we also performed complementary analysis using k = 3 (67%), k = 2 (50%), and k = 3 inverting the training part size (33%) and k = 10 also using the smaller part (10%) for training. This procedure can furnish an important understanding of how much information is sufficient to obtain a satisfactory calibration result. However, increasing the quantity of training data does not always guarantee improved performance of the algorithm. The key factor at this stage is to ensure a statistically representative dataset. Fine-tuning of the hyperparameters, such as the number of estimators, parallel jobs, and criterion, did not present significant accuracy enhancements. Yet, we did not discard that a deep exploratory study on hyperparameter fine-tuning might find a configuration that enhances the results.

The performance of the models was assessed using statistical parameters, obtained using the algorithm output and the reference test data. These parameters were the averaged mean absolute error (MAE), defined in Equation (1), where *y_i_* is the *i_th_* target (reference) output and ${\hat{y}}_{i}$ is the *i_th_* algorithm-predicted output for the given *i_th_* input—

(1) $$\mathrm{MAE}=\frac{\sum_{i=1}^{N}|y_{i}-{\hat{y}}_{i}|}{N}$$

—and the root mean squared error (RMSE), defined in Equation (2), with the same variable definitions as MAE—

(2) $$\mathrm{RMSE}=\sqrt{\frac{\sum_{i=1}^{N}(y_{i}-{\hat{y}}_{i})²}{N}}$$

—the Pearson correlation coefficient (r), given by Equation (3), where $\bar{y}$ is the average of the reference output data and $\bar{\hat{y}}$ is the average of the algorithm predicted output data—

(3) $$r=\frac{\sum_{i=1}^{N}\left(y_{i}-\bar{y}\right)\cdot \left({\hat{y}}_{i}-\bar{\hat{y}}\right)}{\sqrt{\sum_{i=1}^{N}\left(y_{i}-\bar{y}\right)²\cdot \left({\hat{y}}_{i}-\bar{\hat{y}}\right)²}}$$

—and Spearman’s rank-order correlation coefficient (ρ), given by Equation (4), where *D_i_* is the difference between each sample rank when ordinated, and *N* is the dataset length:

(4) $$\rho =1-\frac{6\sum_{i=1}^{N}D_{i}^{2}}{N\left(N^{2}-1\right)}$$

For comparison purposes, the results of the algorithm were put side-by-side with those obtained via simple linear regression for NDIR sensors and negative non-linear relationships for MOS sensors (in the indoor experiment). Perfect models would present values equal to zero for MAE and RMSE, and equal to one for Pearson and Spearman’s coefficients. It should also be emphasized that the output of the MOS sensors is analogue, meaning that their readings were obtained in raw values of voltage. To compare their output in raw conditions to the reference data, which is in the parts-per-million unit (ppm), the nominal conversion curve provided by the manufacturer was used. However, in a preliminary analysis, this conversion curve resulted in inconsistent concentration levels, leading to the observance of very high numerical error values (>1000 ppm). For this reason, further in the Results and Analysis section, we will consider MOS sensors to be “not applicable” for accuracy evaluation in raw conditions.

## 3. Results and Analysis

Considering that our approach was applied in two different scenarios—indoor short term and outdoor long term—we split this section into two subsections, one for each scenario.

### 3.1. Indoors Experiment

After joining the raw data from the indoor exposure experiments, the k-fold split method was used to randomly extract points from each sensor dataset and create modelling subsets. Each subset for modelling the curve equation was formed with 20% of the respective sensor dataset, sampled randomly along the time series. We had not specified seeds in the split process to enhance the randomness and avoid eventual unbalanced sampling to be repeated in all sensor units.

**Figure 5:**
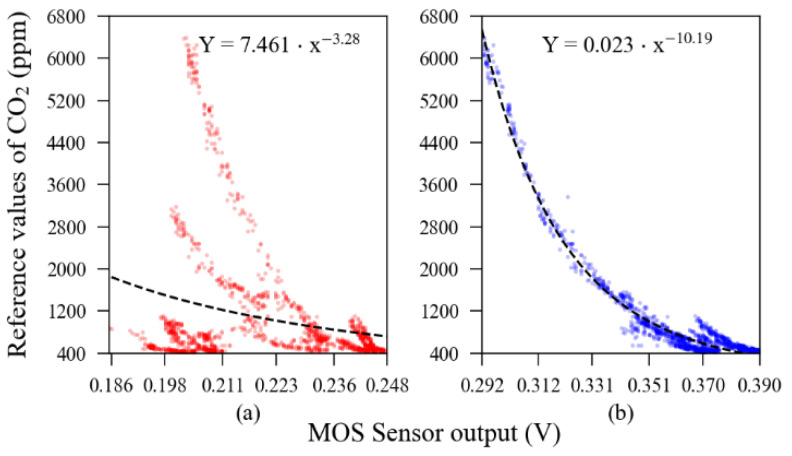
Relationship between raw readings from MOS sensors (unit #1 (**a**) and unit #2 (**b**)) and the reference readings.

**Figure 6:**
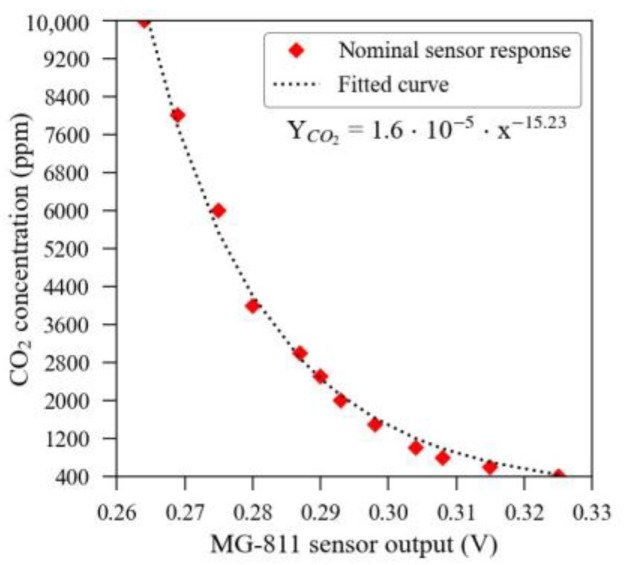
The theoretical response curve of MOS sensors, as provided by the manufacturer.

The MOS sensors’ response and curve fitting model is presented in Figure 5, whilst the theoretical curve, obtained from the manufacturer datasheet, is illustrated in Figure 6. In these figures, one can notice a significant discrepancy between the theoretical response and the experimentally observed output that prevented these sensors from being analysed in a raw condition.

**Figure 7:**
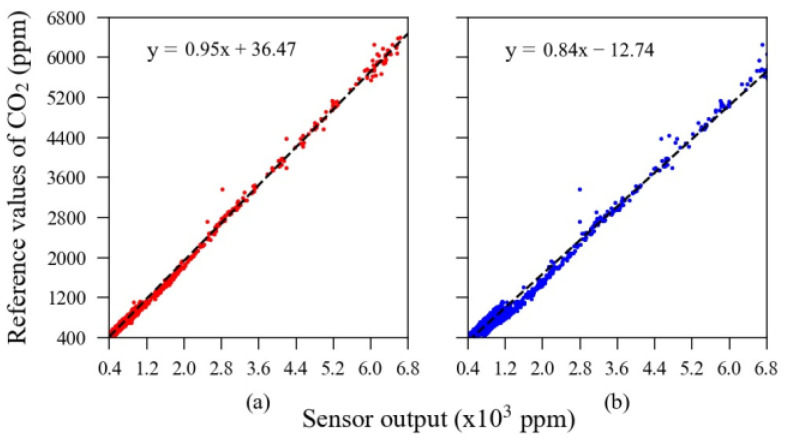
Raw relationship obtained via the NDIR sensors’ readings: (**a**) unit #1; (**b**) unit #2.

**Figure 8:**
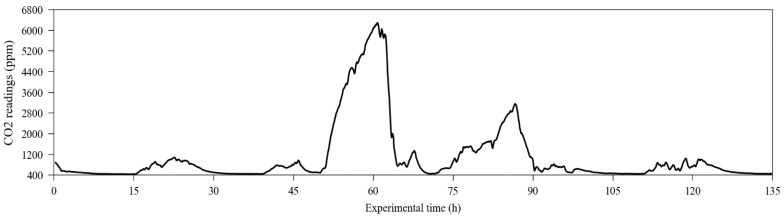
Readings for carbon dioxide were obtained from the reference instrument in the exposure experiments.

The NDIR sensors, which were previously calibrated using their automatic one-point calibration feature, used in fresh air to detect the 400 ppm level, have their raw response curve (also using 20% of the dataset, randomly chosen by the algorithm) depicted in Figure 7. The time series containing the carbon dioxide readings from the reference instrument, used as the target dataset, is presented in Figure 8.

**Figure 9:**
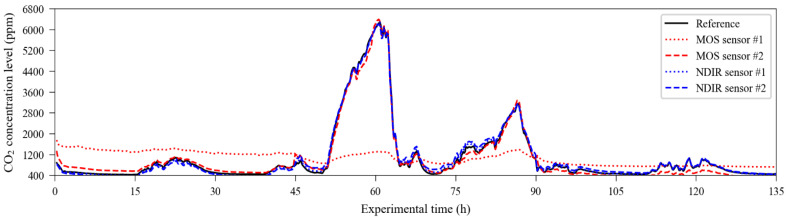
Individual sensor output time series obtained from the extrapolation of the conversion equations.

From this information, it was possible to compare the performance of the raw readings of the sensors with the performance obtained through calibrating the dataset using the response curve obtained from the modelling subsets. The numerical results of this comparison are presented in Table 1. In terms of accuracy, direct calibration achieved good results for NDIR unit 1, moderate results for NDIR unit 2 and MOS unit 2, and poor results for MOS unit 1, which presented a hysteresis-like response. The time series containing the sensor readings adjusted using the extrapolation of the conversion equations to the entire dataset are presented in Figure 9.

Although one-dimensional direct calibration could make most sensors achieve moderate to good accuracy, this technique may be insufficient to meet the requirements of some applications. In the case of MOS sensor unit #1, the direct calibration curve was insufficient to even extract any useful information from its raw readings.

As performed in direct calibration and nominal conversion curves, we used 20% of the dataset for training the ExtRa-Trees algorithm using k-fold with k = 5 and picking the smallest part as the training subset. As the algorithm is repeated “*k*” times, each one using a new subset picked randomly. In each execution, we calculated the statistical parameters between the reference and the validation subset and stored them to calculate the average. The sensor performance obtained using the machine learning model is presented in Table 2.

**Figure 10:**
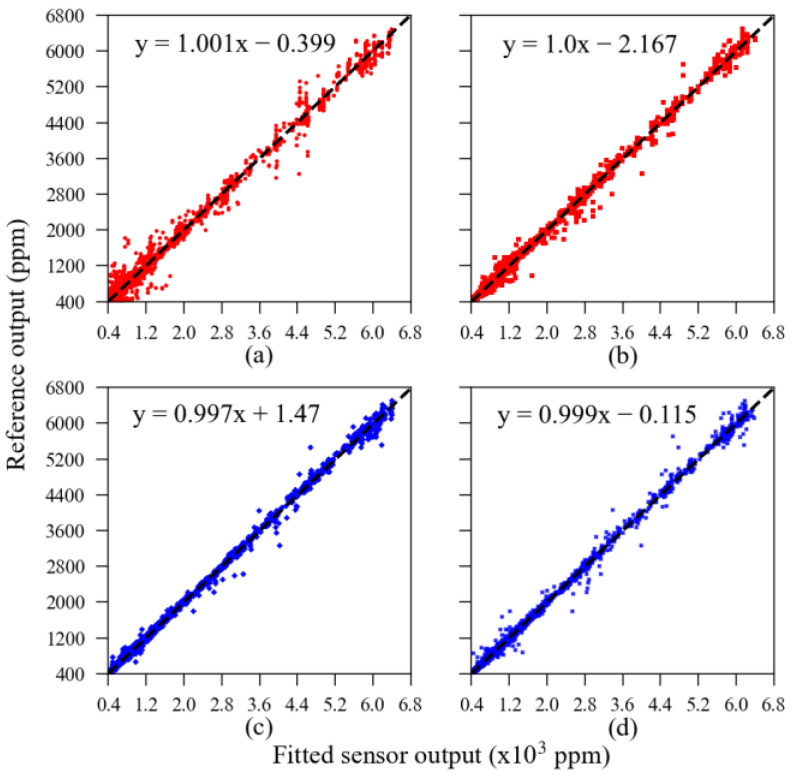
Validation subset calibrated using the ML model. The sensor units are as follows: (**a**) MOS #1; (**b**) MOS #2; (**c**) NDIR #1; (**d**) NDIR #2.

The linear relationships between each sensor unit’s validation subset calibrated using the machine learning algorithm and the reference output are presented in Figure 10. All sensors presented very high linearity after calibration. The residual error scattered throughout the dashed dark line is a visualization of the error’s parameters presented in Table 2. For example, in Figure 10, one can note that the red dots, representing the MOS sensors, are more scattered than the blue dots, representing the NDIR sensors; that is, the overall error observed in MOS sensors’ output was higher than in NDIR sensors’ output.

**Figure 11:**
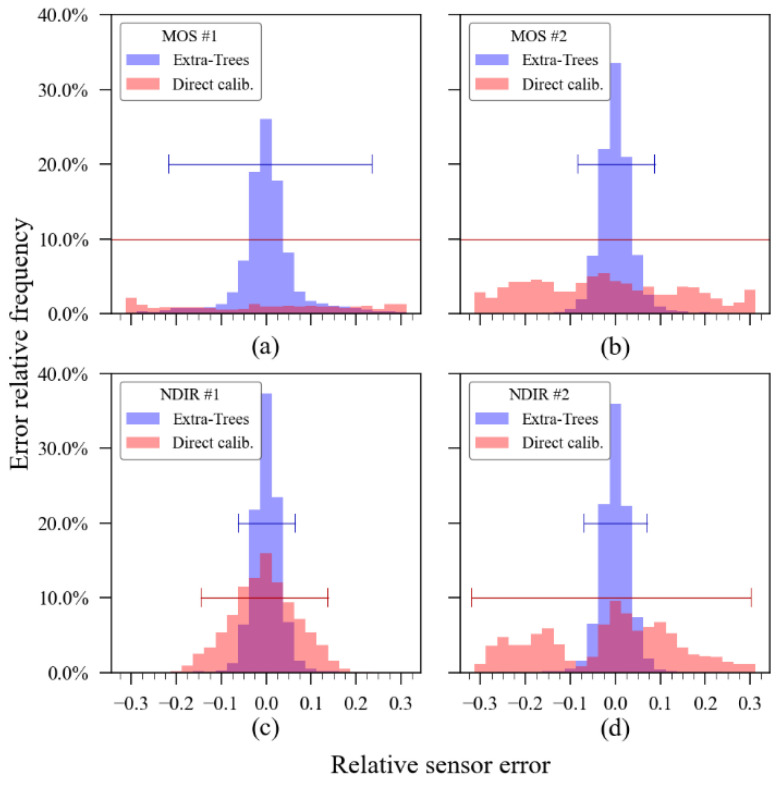
Relative error distribution with confidence interval containing 95% of samples: (**a**) MOS #1; (**b**) MOS #2; (**c**) NDIR #1; (**d**) NDIR #2.

Figure 11, in turn, shows a histogram comparison of the performance achieved using the ExtRa-Trees algorithm versus the performance achieved using the unidimensional curve calibration model. The graphs were built using the relative error of each sensor and each bin has its width corresponding to 2.5% of the relative sensor error. The horizontal lines annotated on the graph correspond to the 95% confidence interval. Note that the MOS sensors with direct calibration showed a confidence interval higher than the axis limits, which means that the unidimensional calibration (annotated as “direct calibration”) for these sensors was ineffective in terms of accuracy enhancement.

From the data presented so far, it is prominent that the most critical performance was observed in the MOS sensor unit #1 data, which presented evidence of significant interference of hysteresis in its readings, making its recovery much slower than it should be when compared to the behaviour observed in MOS sensor unit #2. However, despite the anomalous pattern in the sensor response, the machine learning model was able to extract useful information from the sensor readings, whilst the single-dimension direct calibration was not.

**Figure 12:**
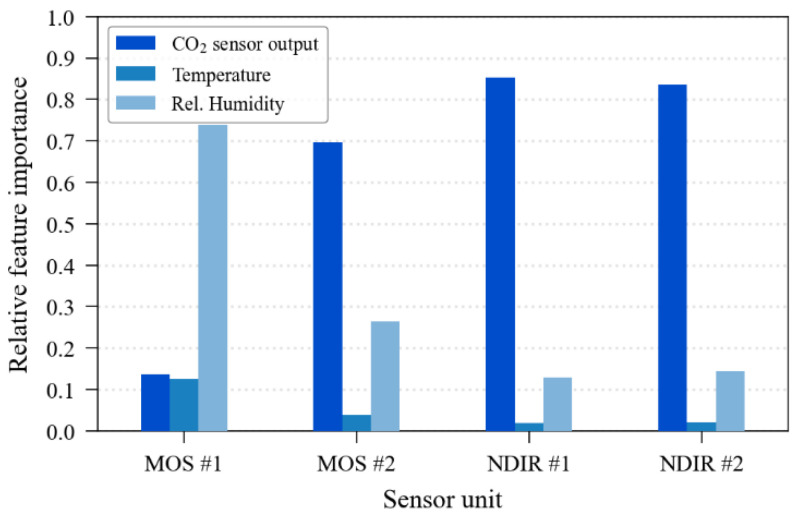
Feature importance for the ExtRa-Trees calibration model.

Figure 12 contains the analysis of the feature’s importance plot, that is, the impact of each input parameter on the algorithm result, in a relative value. One can observe that to calibrate the MOS sensor unit #1, the algorithm resorted much more to the relative humidity data than to the output of the sensor itself. This fact can be an indicator of failure in some internal structures of the sensor since the temperature also showed significant relevance for this sensor (about 12% of relative importance), whilst for the other sensors, it had no explanatory role (<5%). The relative humidity also mildly affected MOS sensor unit #2, as it was found that this feature had about 27% of relative importance in the calibration of this sensor and had a lesser explanatory role for NDIR sensors, with a relative importance of around 13% and 15%. The recurrent observation of this graphic on a periodic calibration scenario for long-term use might be useful to identify the sensor health since the register of the features’ importance history would provide an “expected pattern” for each sensor unit and, consequently, variations on the sensor’s feature importance pattern could be a signal of ageing or incoming failure (e.g., a decrease in sensor output importance along time, such as that observed in the MOS sensor unit #1).

**Figure 13:**
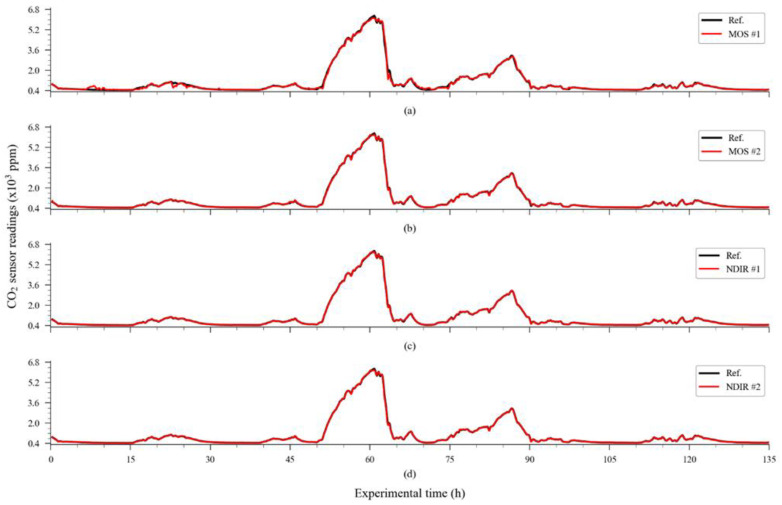
Timeseries obtained via the extrapolation of the ExtRa-Trees model to the entire dataset of each sensor unit overlaid to the reference output: (**a**) MOS sensor unit 1; (**b**) MOS sensor unit 2; (**c**) NDIR sensor unit 1; (**d**) NDIR sensor unit 2.

The accuracy enhancement obtained through using the ExtRa-Trees ensemble regressor can be visualized in detail in Figure 13, which details the time series containing the extrapolation of the model obtained with the training subset to the entire dataset of each evaluated sensor.

**Figure 14:**
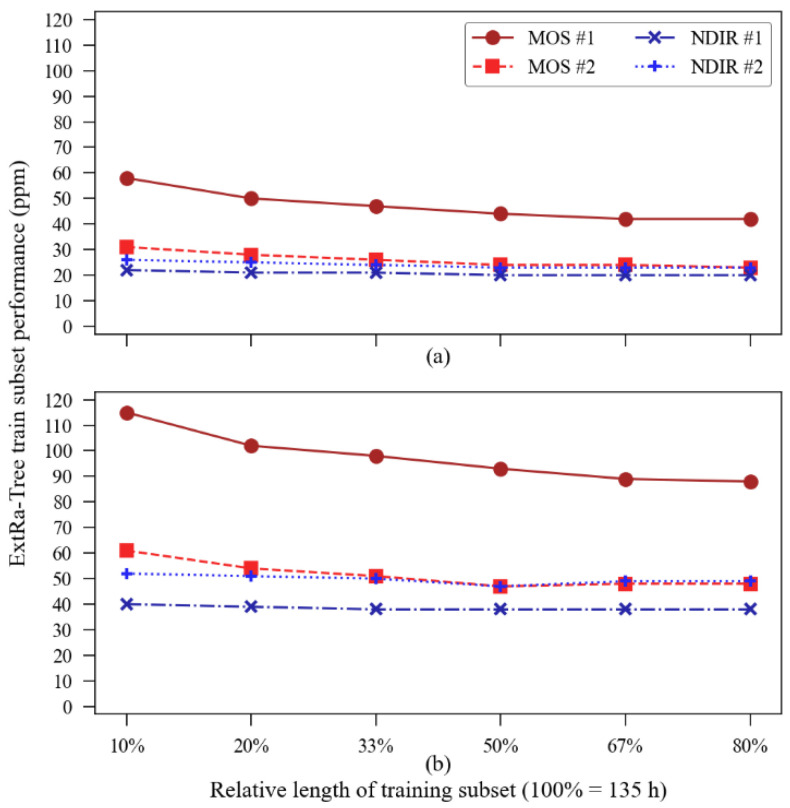
Influence of the training data subset length on the accuracy metrics obtained via the model in the validation subset: (**a**) mean absolute error; (**b**) root mean squared error.

Although the satisfactory results presented so far were obtained from a training subset containing about 27 h of data (20% of 135 h in total) randomly picked from the original dataset and tested into a 108 h dataset built with the points left out of training, we wanted to know the accuracy performance’s dependence on the training subset size. Given this, we conducted an additional analysis to verify the accuracy of the model with different training subset sizes. We intended to investigate how much better (or worse) the results could be with different training sub-dataset sizes. The summary of the training-subset size influence on the accuracy metrics is illustrated in Figure 14.

The MOS sensors presented the most accentuated improvement in accuracy with a longer training subset: MOS sensor unit #1 reduced its MAE from 58 ppm at 10% of subset length to 42 ppm at 80% of subset length and reduced its RMSE from 115 ppm to 88 ppm, while MOS unit #2 reduced its MAE from 31 to 23 and its RMSE from 61 to 48 within the same interval. The calibration of the NDIR sensors showed to be less influenced by the size of the training subset since the highest variation observed in MAE was with sensor unit #2, which has its value reduced from 26 ppm to 23 ppm. Figure 14 also suggests that there is a saturation point in the “training subset size vs. accuracy improvement” relationship, a phenomenon noted by the authors in [41,42]. Direct calibration through the regression function showed no influence of training subset size variations on its accuracy metrics.

### 3.2. Outdoors Experiment

In the dataset resulting from the outdoor exposure experiment, the outputs from the fourteen carbon dioxide sensors were already synchronized with the reference outputs. However, intermittency in data continuity for all sensors was observed and, for this reason, it was necessary to apply an intersection between the reference station data and each sensor’s data to ensure that the machine learning model would receive only valid readings either in its input or target data. Moreover, a new feature was created in the sensors’ timestamps dataset. This new information contains the total exposure time of each sensor in days fractions. Its goal is to verify if the algorithm can identify and compensate for the ageing drift of the sensors, considering that the reference was being periodically recalibrated and, thus, was not influenced by ageing.

**Figure 15:**
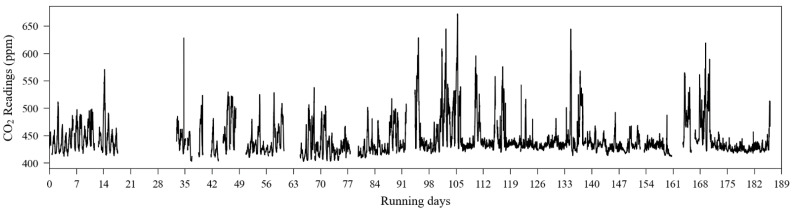
Carbon dioxide reference sensor readings of the outdoor experiment conducted between August 2016 and February 2017.

**Figure 16:**
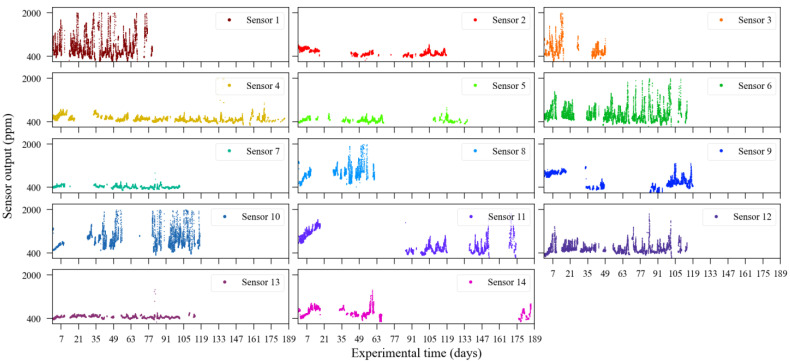
Carbon dioxide low-cost sensor readings of the outdoor experiment conducted between August 2016 and February 2017.

The reference sensor output is illustrated in Figure 15. As the experiment was conducted outdoors, carbon dioxide sources have relatively less impact on the gas concentration level in the air, unlike what happens in the indoor scenario. Thus, during the observation period (approximately 186 days), and according to the reference data, CO_2_ concentration levels were contained between 400 ppm and 700 ppm. The individual outputs from the 14 CO_2_ sensors are shown in Figure 16. In both Figure 15 and Figure 16, it is possible to identify discontinuity in the sensor readings. The biggest data gaps on each sensor time series were caused due to the rotation of sensor deployment during the monitoring campaign since not all sensors were collocated at the same time.

A simple visual analysis of Figure 16 allows one to note that not all evaluated sensors provided their readings according to the values observed by the reference instrument (Figure 15). As an example, it can be cited that the sensors units 1, 6, 8, and 10 presented readings reaching 2000 ppm, the sensor’s maximum range.

**Figure 17:**
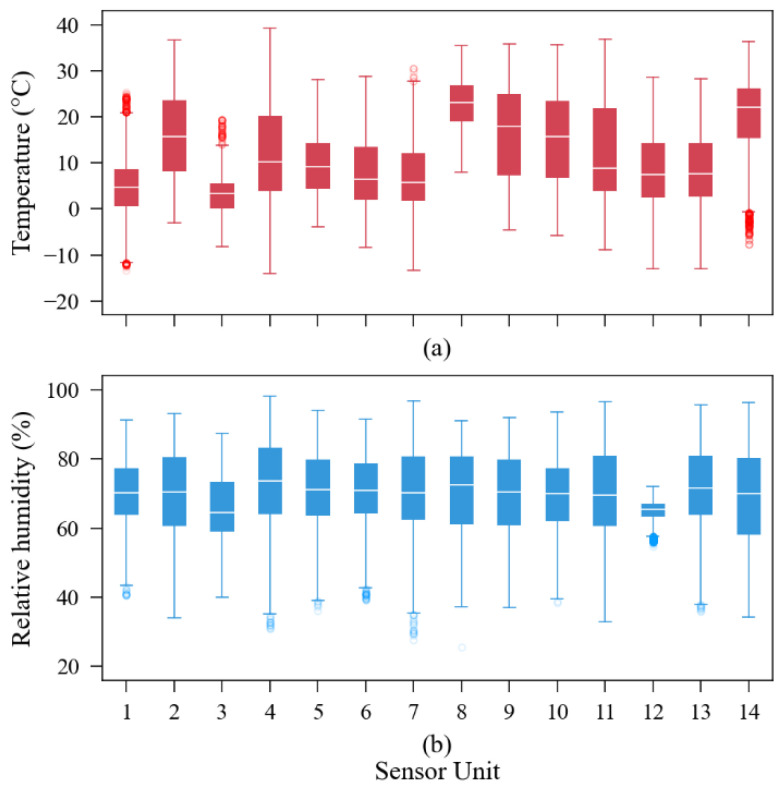
Boxplot of all (**a**) temperature and (**b**) humidity readings of the fourteen sensor units evaluated in the third-party dataset.

To prepare each sensor dataset to be used by the algorithm, it was necessary to overlay the data availability of each sensor unit with the reference sensor through its timestamps, avoiding using input data without target data, or vice versa (see Figure 3). As the SST CO_2_S-A sensor also provides temperature and relative humidity readings together with the carbon dioxide readings, it was not necessary to take any additional action towards these features regarding data preparation. All the temperature and relative humidity readings of the fourteen sensors are summarized in Figure 17. Some sensors were exposed to higher environmental variations than others, for example, sensor unit 4 versus sensor unit 8 in terms of temperature, or sensor unit 4 versus sensor unit 12 in terms of relative humidity.

To guarantee the possibility of a comparative view of the performance of these sensors before and after the use of the ExtRa-Trees algorithm, the accuracy metrics of these sensors were calculated both in raw conditions and after a linear regression calibration, obtained with 20% of each sensor dataset, randomly chosen using the split process as described further, in the machine learning paragraph. The numerical performance of this comparison is given in Table 3.

Although the linear regression was able to numerically reduce the mean absolute error (MAE) and the root mean squared error (RMSE), this fact is not satisfactory per se in terms of accuracy enhancement because both the Pearson and Spearman’s rank correlation resulted in low values for all sensors, suggesting that the linear regression will not suffice for reaching higher accuracy with these sensors. Some sensors demonstrated a very low degree of correlation with the reference instrument, with the most critical cases being observed in sensor units 8 and 11, which had a Pearson correlation coefficient of 0.160 and 0.151, respectively. This suggests that the reduction of the error parameters of these sensors could also be achieved through simply applying an average over their readings and correcting its bias. Moreover, except for sensor units 5 and 7, all sensor units also presented a low degree of correlation (r < 0.35) even after the linear regression adjustment. An important finding after this analysis is that a single-dimensional direct calibration, such as linear regression, was not efficient enough to achieve higher accuracy using this low-cost sensor model in outdoor exposure.

**Figure 18:**
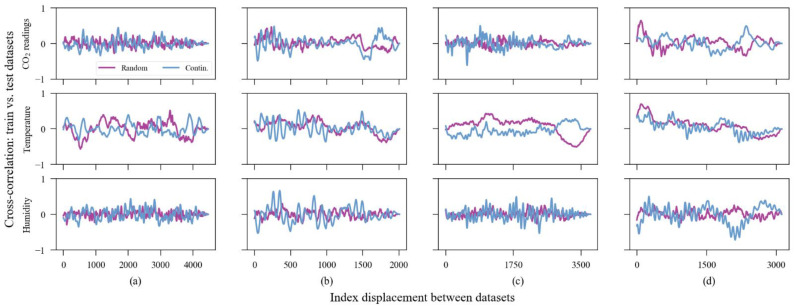
Cross-correlation between train and test subsets. In purple, the training/test relation is 20/80 and data were collected randomly; in blue, the training/test relation is 80/20 and data were collected continuously. (**a**) Sensor #1, (**b**) Sensor #8, (**c**) Sensor #10, (**d**) Sensor #14.

Regarding the machine learning algorithm, the data split process which separates the datasets between training and validation data subsets was designed differently from the common way. Although the experimental data are limited in time, these sensors were exposed outdoors for a significant time and we noted that the input parameters were not homogeneously distributed over the observation time. We assume the uncalibrated sensor error is a function of four variables: carbon dioxide, air temperature, relative humidity, and exposure time. Using, thus, a continuous range of data to train the model may not include all combinations of climatic variables existing throughout the dataset (e.g., imagine a calibration model being trained with data collected during winter and being tested over data gathered during spring). To circumvent this heterogeneous distribution of input parameters, the collection of points used for training the calibration models occurred randomly throughout the entire dataset. Aware of the risk of implying a dependency between training and test data, we reduced the size of the training dataset to 20% of the total to avoid creating two correlated datasets. Figure 18 demonstrates the cross-correlation between training and test subsets in two situations: the training dataset created with 20% of random points and when the training dataset was created with 80% of continuous points from the beginning of the time series. Considering that there was no consistent strong correlation between the datasets, we assume that we created an independent dataset for testing the model. However, some spurious correlations appear occasionally. We assume that this occurred due to the periodicity of the weather. Moreover, from this point on, we have chosen to present only the data of four sensor units to keep data visualization clearer, reducing the number of subplots and yet ensuring a closer look into the graphics. The sensors chosen for visualization were units 1, 8, 10, and 14. They were chosen randomly between those that presented the highest MAE values in raw conditions (>100 ppm). Those are the sensors with the most critical accuracy issues.

**Figure 19:**
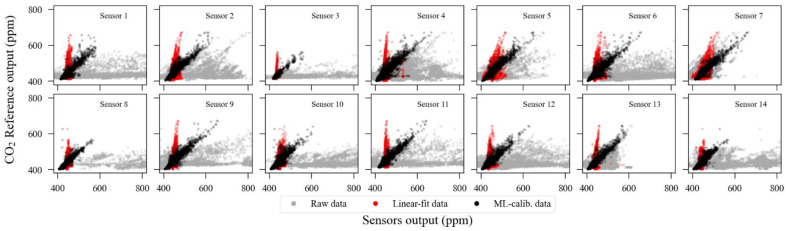
Scatter plot of SST CO_2_S-A sensors’ data from the outdoor exposure experiment in three different formats: raw (grey), linear regression (red), and machine-learning multivariate calibration (black). The axis limits were truncated to 800 ppm for better visualization.

Given the described split method, we used k-fold with k = 5 to test five different combinations. Considering that each sensor has a different continuous exposition time, the training subset size, in absolute time, may differ between sensor units. The algorithm is repeated k times, each one using new data points for training, and the presented metrics are an average of these executions. As mentioned in the indoor experiment analysis, we have performed no additional fine-tuning on the algorithm hyperparameters once the results obtained with its default configuration could be considered satisfactory. However, we do not discard that a deeper investigation on this topic could enhance, even slightly, the accuracy metrics. Figure 19 contains the scatter plot between all sensors in all conditions versus the reference: raw, linear regression, and ML-calibrated. Table 4, in turn, presents the metrics achieved with the ExtRa-Trees algorithm using k = 5 in the k-Fold split process.

Sensor units 8 and 11 in the uncalibrated (raw) dataset presented the lowest correlation degrees with the reference, with r-values of 0.16 and 0.15, respectively. However, after the calibration with the machine learning model, these sensor units reached the r values of 0.913 and 0.948, respectively. In simple terms, the algorithm was efficient enough to transform the poor-quality and nearly uncorrelated readings of these sensors into meaningful information with a very strong degree of correlation with the reference instrument. The highest Pearson correlation after the machine learning calibration was observed in sensor unit 7, with an r-value of 0.96. When uncalibrated, the same sensor presented an r-value of 0.525.

**Figure 20:**
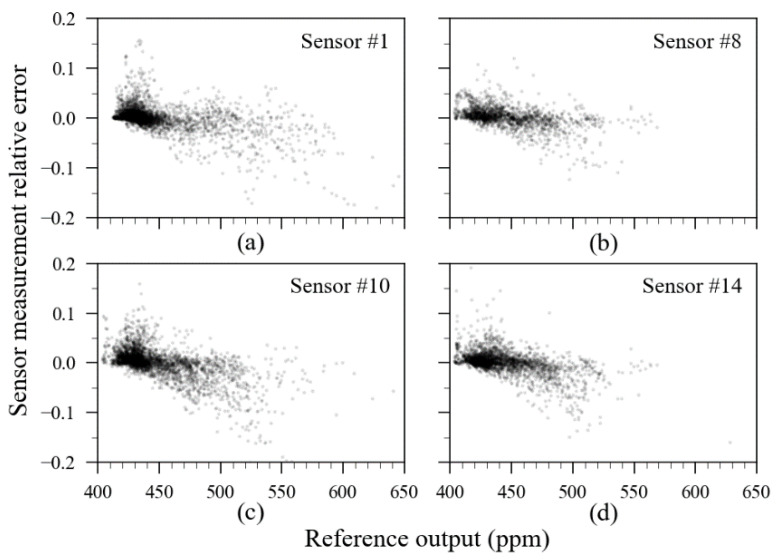
Reference readings versus sensor measurement relative error of the example sensors after ML calibration. In order: (**a**) sensor unit 1; (**b**) sensor unit 8; (**c**) sensor unit 10; (**d**) sensor unit 14.

Although the numerical parameters presented in Table 4 together with the information contained in Figure 19 can transmit a good idea about the sensor accuracy, a graphic dedicated to visualizing the distribution of the relative errors after ML calibration along with the measurements is necessary for enhanced analysis. The relative error plot of these sensors is presented in Figure 20.

Transparency was applied in the data points presented in Figure 20 to create a density-like plot. A darker region means more readings. On this, it is noticeable that the highest density of readings is contained in the 400 to 500 ppm range. Relative errors beyond the ±5% range are rare; however, they are visually more noticeable when CO_2_ concentration levels increase.

**Figure 21:**
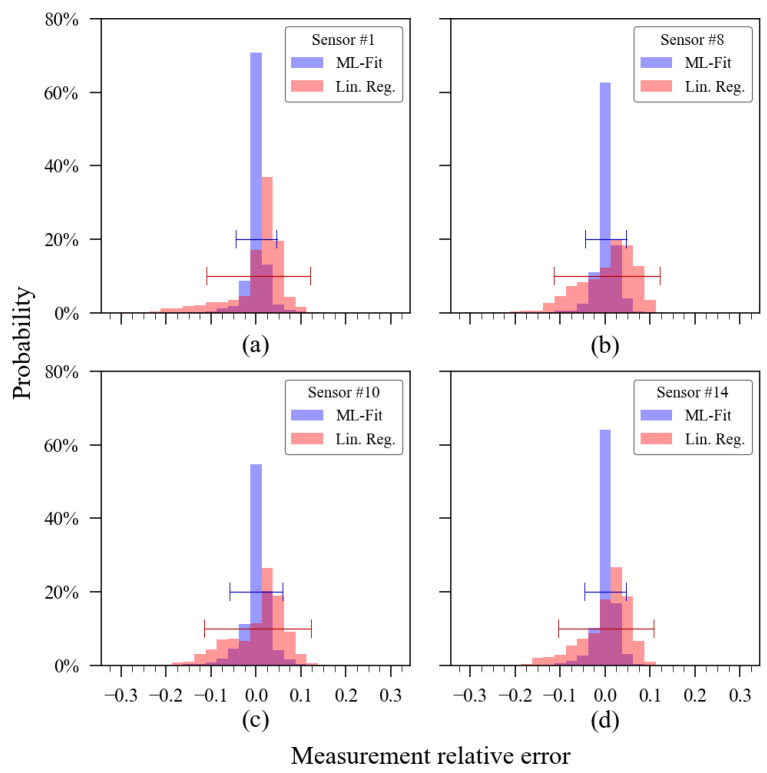
Histogram of relative error from sensor units 1 (**a**), 8 (**b**), 10 (**c**), and 14 (**d**). Machine learning validation data are in blue, whilst the linear regression calibration is overlaid in red. blue and red bars comprehend the 95% interval limit for each data.

Figure 21 presents histograms of the relative errors extracted from the validation dataset of these sensor units. For comparison purposes, the relative error histogram of the validation dataset obtained via linear regression for the same sensor units was also added to the plots.

**Figure 22:**
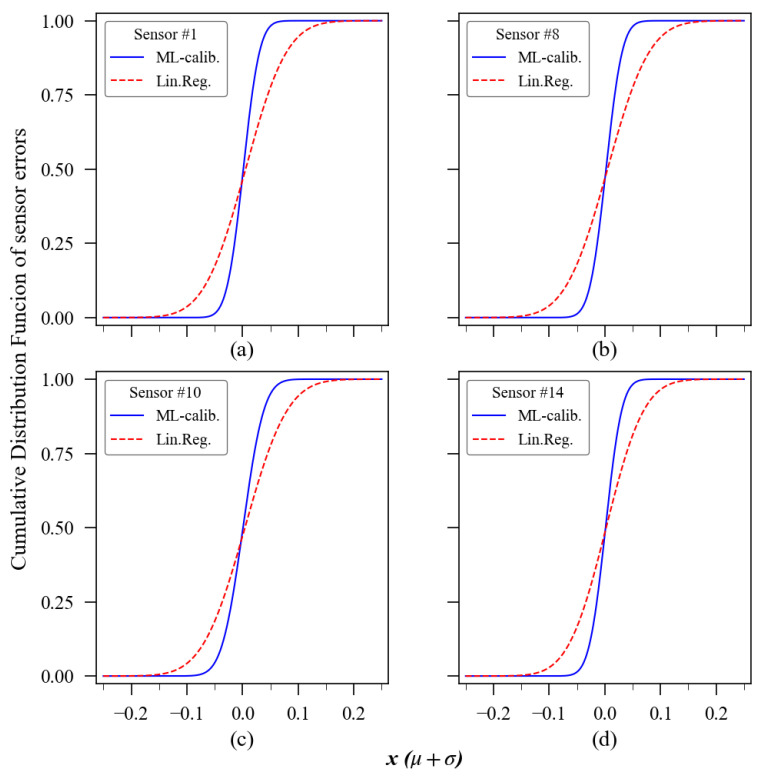
Cumulative distribution function of the relative error of (**a**) sensor unit 1; (**b**) sensor unit 8; (**c**) sensor unit 10; (**d**) sensor unit 14.

In the relative error histogram (Figure 19), each bin has a width of 0.025 (2.5% of measurement relative error). Comparing the shape formed by the red and blue bars on it, one can have a better understanding of the accuracy enhancement achieved using the ExtRa-Trees model individually trained for each sensor unit. Choosing sensor 1 to exemplify, when using linear regression, 95% of its relative errors were contained in the ±12.5% margin, whilst after ML calibration, the 95% interval was delimited between the ±4.6% range of relative error. The other sensors’ confidence interval (95%) of the relative measurement error also showed satisfactory results. For sensor units 2 to 14, the 95% confidence interval was, respectively, ±4.4%, ±2.4%, ±5.1%, ±4.6%, ±5.2%, ±3.9%, ±4.6%, ±4.8%, ±5.8%, ±4.2%, ±3.9%, ±3.6%, and ±4.6%. The theoretical cumulative distribution function (CDF) of the relative error, complementing this histogram, is presented in Figure 22, which also includes the parameters from the linear regression calibration to compare the performance with the machine learning model results. The ideal result for such a graphic would be a step-like curve, with the transition nearly vertical.

**Figure 23:**
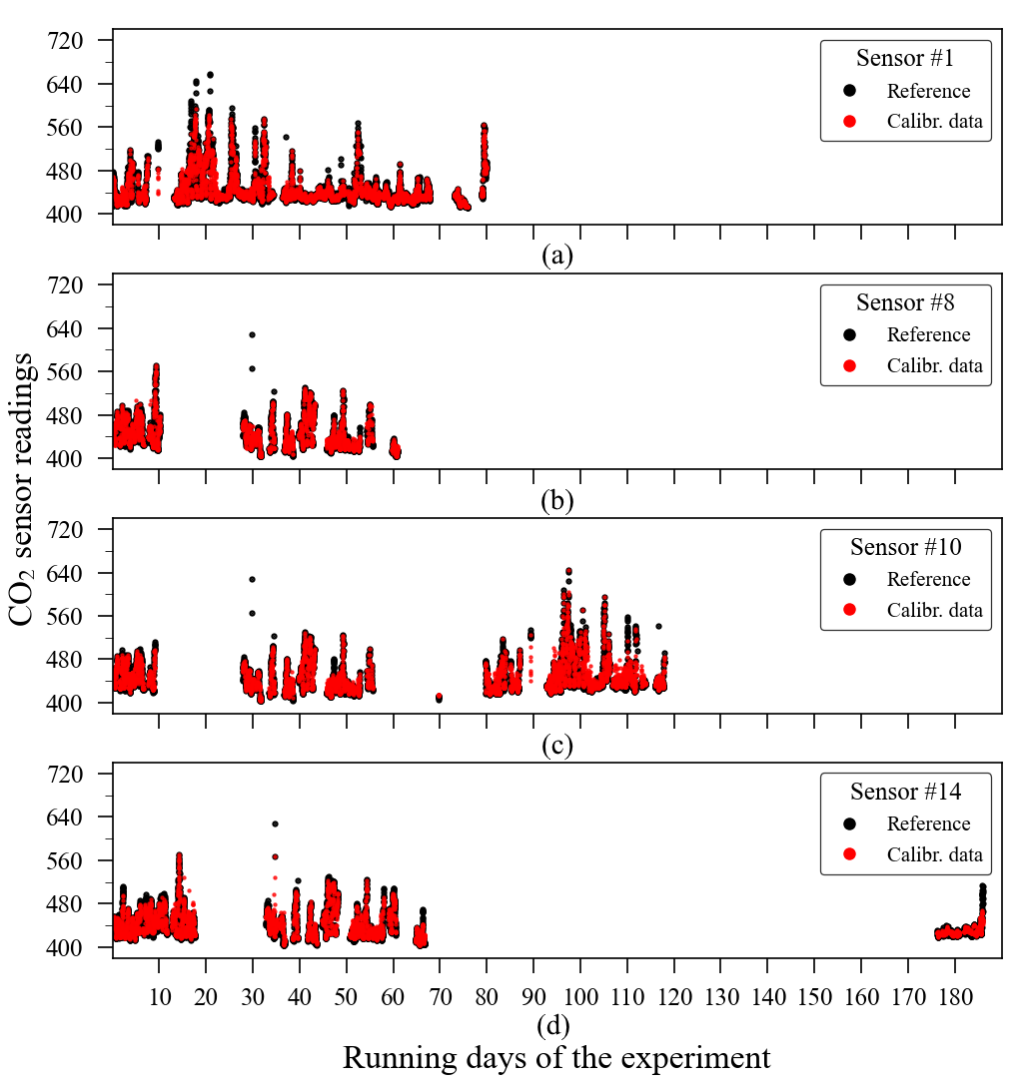
Time series of individual sensor units calibrated with the machine learning ExtRa-Trees model: (**a**) sensor unit 1; (**b**) sensor unit 8; (**c**) sensor unit 10; (**d**) sensor unit 14.

The time series of the calibration model on the example sensors are presented in Figure 23. This image allows one to identify where and when the discrepancies between the reference and the calibrated data exactly happened. It is important to emphasize that this image contains only data points from when both reference and the evaluated sensors’ readings were available at the same time.

**Figure 24:**
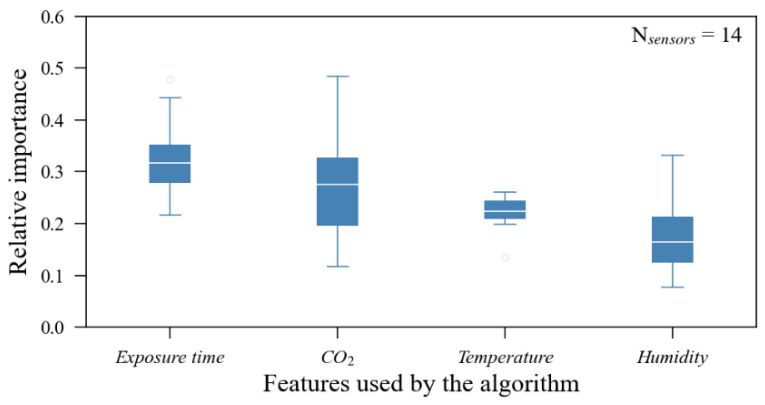
Feature importance boxplot built from a compilation of all (14) individual calibration models.

The ExtRa-Trees calibration model is an ensemble regressor that does not perform its prediction via a weighted equation using multiple input parameters but rather using estimators and decision nodes based on each input parameter value. Given this, it is possible to visualize the relative relevance of each input parameter for sensor error reduction. Figure 24 presents a boxplot created through the aggregation of all features’ importance extracted from the individual calibration models.

**Figure 25:**
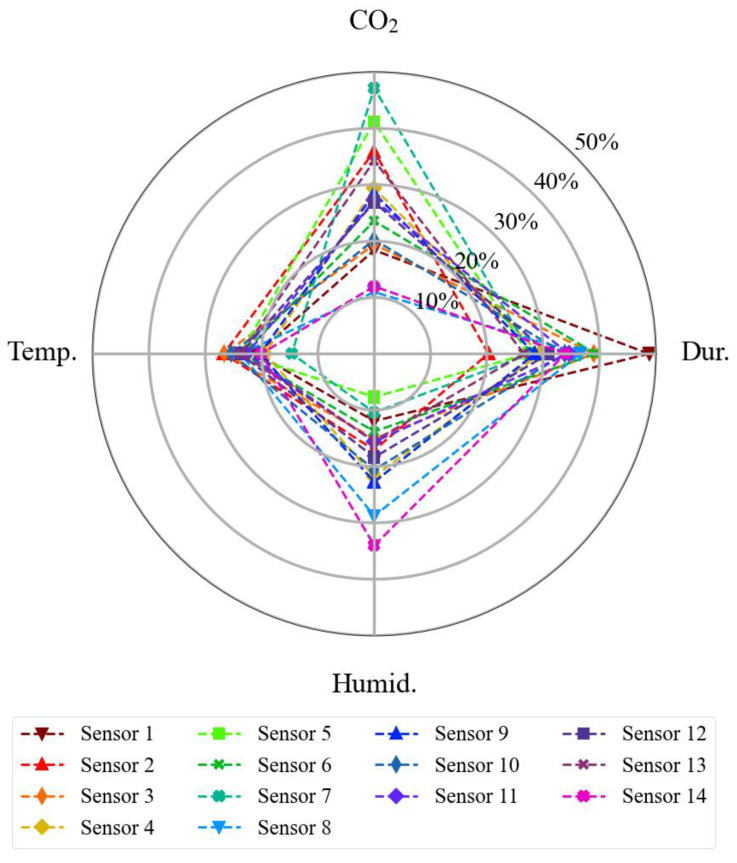
Radar plot containing the importance of the features from each ExtRa-Trees calibration model.

All input features presented a relevant explanatory role. The parameter that showed the highest variation in its relevance between the models was the CO_2_ output, whilst the least variation was observed in the temperature parameter. In general terms, the average relative relevance was 32.5% of exposition time (minimum of 21%, in sensor #2; maximum of 47.9%, in sensor #1); 27.1% of CO_2_ sensor output, in ppm (minimum of 11.1% in sensor #8; maximum of 47.7% in sensor #7); 22.2% of temperature readings (minimum of 13.7% in sensor #7; maximum of 26.7% in sensor #2); 18.2% of humidity (minimum of 7.9% in sensor #5; maximum of 34.3% in sensor #14). A composition of all features’ relative importance resulting from all calibration models is illustrated in Figure 25.

Additional verification of the algorithm’s performance must be performed to ensure whether the importance of the features was a result of a false positive or not. A feature with falsely high importance is common in data with high cardinality [43]. From the input parameters used, the exposition time is the feature that presents the highest cardinality, since all values are unique (each value is a fraction of a day). The most efficient way to perform this check is to repeat the algorithm’s training process without the sensor exposition time as an input variable and then recalculate the accuracy metrics obtained to compare with those achieved using the algorithm trained with the exposition time included. In the case of significative depreciation of performance metrics parameters (MAE, RMSE, and r), it can be concluded that the variable in question was truly significant in reducing the error. On this, it was observed that the exposition time was, in fact, relevant for the algorithm to optimize error reduction, considering that the removal of this variable from the input features yielded depreciation in the numerical performance parameters calculated from the same validation subset, as illustrated in Table 5 (in comparison to the values presented previously in Table 4). That means the algorithm could identify the sensor ageing within the given period. This influence, in turn, was more accentuated in some sensor units than in others.

Regarding the mean absolute error, the sensors presented an increase of up to 100% (minimum of 33% in sensor unit 12; maximum of 100% in sensor units 1, 3, and 7). In terms of the root mean square, the maximum increase observed was 75% in sensor unit 1, whilst the minimum increase occurred in sensor unit 10 (33%). Sensor units 10 and 12 seemed to suffer less influence from ageing than the other units. Another appropriate analysis to consolidate the importance of using the continuous exposure time of the sensors in the algorithm’s training stage is to compare the confidence interval of the relative error obtained in the test subset in both situations: with and without the use of this variable. Table 6 shows the confidence interval (95%) of relative errors of the sensors’ dataset calibrated using the ExtRa-Trees model with (as seen in Figure 20) and without sensor exposition time as an input parameter, as well as the confidence interval achieved via the linear-regression calibration.

The information presented in Table 6 suggests that the use of the continuous exposure time of the sensor might be an important input parameter in machine learning algorithms for error reduction in long terms measurements.

**Figure 26:**
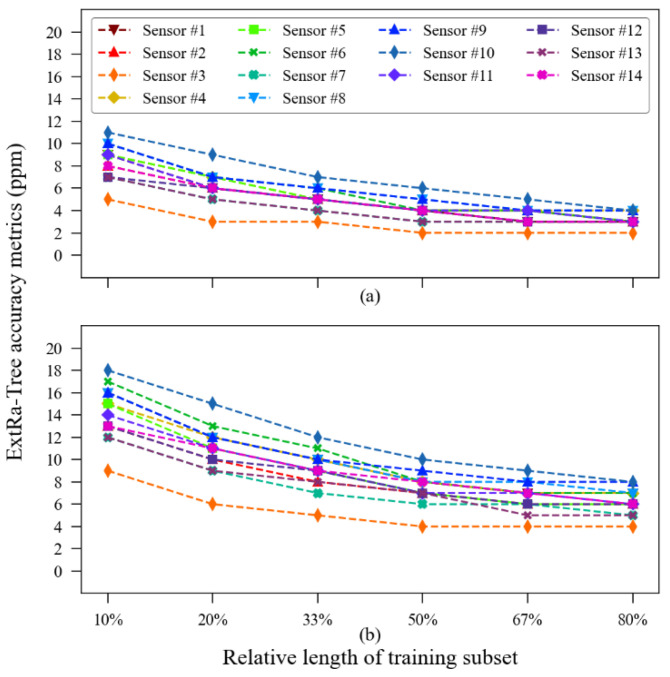
Training subset size influence on accuracy metrics of the ExtRa-Trees model: (**a**) mean absolute error; (**b**) root mean squared error.

Although the achieved results can be considered satisfactory, they were calculated using the training/testing dataset split relation of 1:4 (20%/80%). As each sensor has its own exposure time, therefore, the absolute sizes of each test subset also differ in length. To ensure that the heterogeneity of the exposure time of the sensors may have interfered with the performance metrics of the algorithm, we calculated the dependence between the total exposition time of the sensors in hours (see Table 3) and the root mean squared error of the sensors (see Table 4). As a result, we identified a p-value greater than 0.4 (r = 0.1987, N = 14), pointing out that there is no statistical significance between the changes observed in the values of these variables. Once the total exposition time of the sensors presented no relation with the obtained accuracy metrics, further analyses were made to assess the influence of the relative size of the training datasets on the model accuracy. Other “training vs. test” subset size relations were tested, as follows: 10/90, 33/67, 50/50, 67/33 and 80/20. To summarize this analysis, the MAE and RMSE were the only parameters considered. The influence of the training vs. testing ratio on accuracy metrics for the evaluated sensors is presented in Figure 26.

It is noted that increasing the training subset’s relative size to, and beyond, 50%, both the MAE and RMSE metrics tend to stabilize the error mitigation rate. The error reduction saturation at larger training datasets might be explained due to an overfitting-like effect since the validation dataset is smaller. This phenomenon was not observed in the indoor experiment due to its short duration and, perhaps, due to the more stable environmental parameters which could be satisfactorily sampled with fewer data points, then reaching the optimal error reduction.

## 4. Discussion

In this section, the quantitative analyses presented are further discussed to extract interpretations either on sensor behaviour or machine learning algorithm performance.

### 4.1. Indoors Experiment

**Figure 27:**
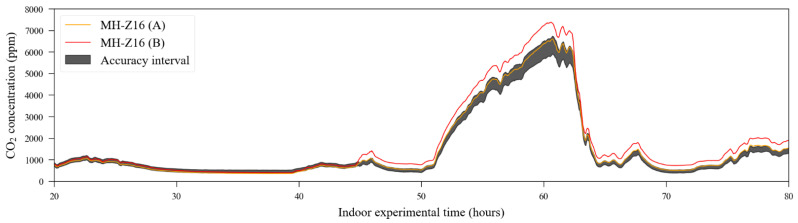
Indoor experiment time series excerpt to show NDIR sensors’ nominal accuracy (in dark grey) and actual readings (in orange and red).

Even though the nominal performance parameters of these sensors appear to be acceptable, in practice, the sensor, when uncalibrated and used in a “plug-and-play” condition, might not comply with its provided specifications. In our experiment, this was confirmed. For example, the MG-811, an MOS sensor, has in its datasheet the statement that it has “low dependence on humidity and temperature”, while the graph of the feature’s importance (Figure 12) demonstrates the opposite for both units evaluated. The nominal response curve of this sensor provided an output completely mismatched with reality. The MH-Z16 (NDIR) sensors, however, can be used satisfactorily in low and mid-end applications despite one sensor unit not reaching the nominal precision in the “plug-and-play” condition (±50 ppm + 5% of the reading for CO_2_ concentrations up to 5000 ppm and ±100 ppm + 5% of the reading for concentrations between 5000 and 10,000 ppm; see Figure 27).

**Figure 28:**
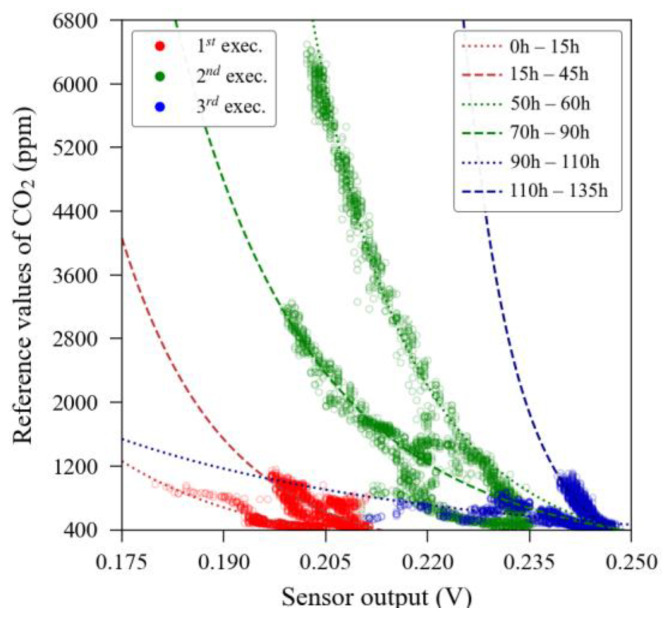
Detailed raw response of MOS sensor unit 1 with experiment pattern identification.

Unidimensional regression, either linear or non-linear, is the first calibration method that the end-user might resort to when a reference instrument is available because it is a straightforward process. We observed that the unidimensional calibration using only the reference as a guide dataset may be able to enhance linearity and reduce errors in significant quantities (up to 60% of MAE reduction, as observed in the validation dataset of NDIR sensor unit 2), but it still keeps a significant error spread, as one can observe in Figure 10, where the best sensor performance—NDIR unit 1—still kept 95% of its relative errors contained around the mark of ±15% (relative to the actual measurement). The MOS sensors presented a much more critical error pattern, with their 95% interval of relative errors exceeding the ±30% mark for both sensors. This occurs, perhaps, due to the significant dependence on climatic variables of the MOS sensors. Moreover, we also observed that the accuracy of direct unidimensional calibration models, whether linear regression or inverse power law relationship, did not change regardless of the variation in the size of the training subset within the evaluated intervals, maintaining the accuracy values presented previously in Table 1 for all training subset sizes. However, this training model was obtained from random points distributed alongside the dataset. This occurred to maintain the most of the sensor characteristics as possible. For example, if the data points for training were collected from a continuous part (e.g., the first 20% of the dataset), the performance of the MOS sensors could deteriorate, because it would lack information contained in the hysteresis-like curve observed when CO_2_ concentrations were high (the MOS sensors presented one curve response when CO_2_ concentrations are rising and a different one when the CO_2_ concentration is diminishing). Moreover, the MOS sensors showed to be more susceptible to environmental interference. Figure 28 illustrates in detail this anomalous behaviour, where each colour corresponds to the data obtained from a different experimental execution. Note that there are six major patterns for this sensor, each one represented by a fitted curve using an inverse power law relationship.

Table 7 depicts the environmental parameters for each period annotated in Figure 26 and its corresponding conversion curve. In a hypothetical situation in which the user would have access to a reference instrument for a limited time window, the calibration of a MOS sensor with this behaviour would be compromised. Moreover, the last two columns of Table 7 exemplify how difficult it would be to calibrate this sensor unit using conventional methods as the coefficients varied significantly during the experiment—even by orders of magnitude—and did not follow any noticeable pattern.

Concerning the ExtRa-Trees algorithm, the ML model was able to remove any systematic error (bias) from the sensors as well as significantly reduce the random error. Thus, the overall accuracy was satisfactorily enhanced, with mean absolute error reductions varying from 53% up to 93%, and RMSE reductions from 33% up to 91%, compared to the one-dimensional regression metrics. A summary of the improvements obtained using the ExtRa-Trees model in the accuracy parameters is shown in Table 8. These results were achieved using the model when trained with about 27 h of data (a relative size of 20% of the entire dataset). An increase in the training subset length, as previously presented in Figure 14, may benefit the MOS sensors’ accuracy enhancement, but there is a saturation point (limited by overfitting). This limit appears to happen around 90 h of training data for MOS sensor unit 1 (2/3 of the total dataset) and around 67 h for MOS sensor unit 2 (1/2 of the total dataset). The NDIR sensors seemed to be at the accuracy enhancement cap, as the training subset length showed no significant increase in the parameters presented in Table 8.

Moreover, the error distribution analysis showed that 95% of errors after ML calibration were contained within the ±10% range, in relative measurement, except for MOS sensor unit 1, with 95% of its relative errors contained within the ±22% range.

To compare the results obtained to a practical example, the Portuguese ordinance 3553A-2013 [44] was taken as an example. It is an official document that tackles air quality standards, which is specified as a protection threshold for individuals with a concentration level of 2250 mg/m^3^ of CO_2_ (equal to 1161 ppm) with 30% tolerance (1509 ppm). Table 9 presents the 95% confidence interval of the relative errors of the sensors considering this stipulated threshold. Most of the sensors demonstrated satisfactory accuracy within the exemplified requirement after calibration with the machine learning algorithm, except for MOS sensor unit 1. Also, the unidimensional calibration (regression) showed smaller relative errors in higher concentrations.

In terms of environmental dependence, disregarding MOS sensor unit 1, the sensors did not show significant interference from temperature within the range observed in the indoor experiments (T_min_ = 20 °C and T_max_ = 25.5 °C), probably due to the sensors’ auto-compensation feature. On the other side, relative humidity, which varied from 17.2% to 60.5%, seemed to affect the sensors more, as the feature importance plot indicated that its relative importance was above 20% for MOS sensors and above 10% for NDIR sensors. This finding suggests that for a high-accuracy application for CO_2_ monitoring using sensors with the technologies evaluated in this experiment (metal oxide semiconductor and non-dispersive infrared), a proper humidity measurement might be required in the vicinity of the carbon dioxide sensor. Concerning MOS sensor unit 1, despite the irregular behaviour of this unit (as seen in Figure 26), probably caused by a malfunction of some internal component, the ML model was able to extract information from its poor-quality outputs through identifying that the relative humidity was the most interfering parameter and compensating for this interference.

The accuracy level obtained after utilization of the ExtRa-Trees model in this experiment may enable these sensors for several applications. As a first example, we may cite the air quality monitoring system for environments where individuals perform productive tasks, such as schools, universities, and offices. In such places, when the air conditioning does not provide air renewal to the climatized environment (very common in tropical climates, where the outdoor air temperature is above 30 °C), carbon dioxide accumulates over time and may affect the productivity of individuals. With a monitoring system, one can simply perform a manual action such as opening the windows or doors when the level is about to trespass the threshold of 1000 ppm.

These sensors can also be applied in research projects that use gas concentration levels to identify related phenomena, such as room occupation [26,27], behaviour monitoring systems [45], and even to assess productivity in apiculture through monitoring honeybees’ parameters [46,47].

Although the ML model has been trained and validated “offline”, that is, using data that were previously collected, there is a possibility of exporting the trained object for execution in real-time calibration. In future work, for example, it can be embedded in a node of an environmental monitoring network, provided that this node has sufficient computational capacities to execute the algorithm or that the algorithm is adapted to the computational capacity of the node.

### 4.2. Outdoors Experiment

The field experiment carried out by Zimmermann et al. [22] used, for carbon dioxide monitoring, the sensor SST-CO_2_A, an NDIR device whose nominal accuracy parameters are ±30 ppm + 3% of the reading, for the version with a measurement scale of 2000 ppm. In terms of the transduction principle, this sensor model uses the same technology as the MH-Z16A evaluated in the indoor experiment.

We observed that even if previously calibrated, the low-cost CO_2_ sensors commonly exceed the theoretical accuracy parameters when put into use, as per the raw metrics parameters indicated in Table 3. Yet, this was less critical on the NDIR sensors evaluated in indoor conditions. Even after linear regression calibration, the sensor units exposed outdoors still presented poor performance, as most of them showed Pearson coefficient values below 0.5 after testing each validation subset against the reference instrument. In the medium/long-term outdoor experiments, the unidimensional calibration is expected to deprecate because there is a greater heterogeneity of the climate and, therefore, varied combinations and unstable conditions of air temperature, relative humidity, and wind incidence. In places with greater climatic variation along seasons (e.g., continental places far from the Equator) or extreme weather, the field deployment of low-cost gas sensors in the collaborative sensing context might be quite challenging, provided that users have limited access to useful resources for sensor data quality enhancement. Beyond that, in a long-term monitoring campaign, there is still a concern regarding sensor ageing, which causes monotonic drift: a slow change over time (increase or decrease) in the base value output of the sensors.

In terms of error reduction achieved using ExtRa-Trees, when comparing the performance presented in Table 4 (accuracy parameters after being calibrated with the machine learning algorithm) to the error parameters in the raw condition (Table 3), reductions were observed in the mean absolute error between 84.8% and 98.8%, depending on the sensor unit. The percentual reduction in the same parameter compared to the dataset calibrated via linear regression was between 60.9% and 75%. The RMSE parameter, which penalizes the highest errors, was reduced using linear regression between 81.6% and 98.4% if compared to the raw dataset, whilst a reduction between 48.3% and 69.7% was achieved using ExtRa-Trees. The error reduction rates of all techniques deployed, compared to the original error against the reference instrument, are summarized in Table 10.

The lower reductions in error achieved by the ExtRa-Trees model on relative terms happened on those sensors that presented the lowest errors in raw conditions (sensor units 4, 5, 7, and 13). The overall MAE result is the averaged value of 6.2 ppm. We did not weight this parameter using each sensor unit exposition time because, as mentioned, no causal relationship was found between the total time of sensor usage and respective error mitigation. If we consider the outcomes reached via the method presented in this investigation when using k = 5 and 80% of each sensor dataset as training, the accuracy achieved using the ExtRa-Trees model is more promising: 3.2 ppm of averaged MAE (2–4 ppm).

For comparison purposes, the calibration model of the experiment’s authors reached an overall error (averaged MAE from all sensor units) of 10 ppm for the carbon dioxide sensors. Moreover, their method was focused on a multi-pollutant platform and used 80% of the dataset as training in k-fold cross-validation (k = 5). They used different gas sensor data as inputs to detect eventual cross-sensitivity. Our method, in turn, was focused only on the carbon dioxide sensor data. Thus, we managed to reach a satisfactory accuracy even after reducing the data complexity through removing variables corresponding to other pollutants (reducing, consequently, the computational cost). In a similar manner, we found that sensor exposure time is also an important variable to be used as an input parameter in the algorithm for long-run calibration, since we managed to reduce the 95% confidence interval of the relative sensor measurement error to the range of ±2.4–5.8%, depending on the sensor unit, whilst this interval, assessed under the same circumstances, was ±7.5–13.3% with linear regression and ±4.4–8.7% with machine learning calibration without considering the exposure time.

Regarding the amount of data used in the algorithm’s training stage, the results achieved by Yamamoto et al. [21] and Zimmerman et al. [22], complemented by the results we managed to achieve, suggest that an important practice in such application is to provide the algorithm with as many input parameter combinations within the operational limits as possible. Passing a larger amount of data, with fewer data combinations, will not suffice to predict new situations. That is, a machine learning model that was trained with sensor data gathered during winter and autumn periods, even in large quantities, would be less effective in calibrating the same sensors during the autumn and summer periods than a model trained with a greater combination of the environmental variables extracted throughout the four seasons, even in smaller quantity. However, without any limitations concerning data availability, the optimal situation would be a model trained with large quantities and yet diverse data. Thus, it is worth noting that the described approach is a post-processing calibration, for short- and mid-term situations where the available data are not enough for comprehensive calibration.

## 5. Conclusions

This paper reported a machine learning calibration procedure and assessment for data obtained from indoor and outdoor experiments using low-cost sensors. The indoor experimental setup exposed four low-cost carbon dioxide sensors, together with a reference instrument and climatic sensors (temperature and humidity), to different concentration levels of carbon dioxide. This setup was run in closed spaces to assess those sensors’ accuracy in raw conditions and then to investigate the limits of accuracy enhancement when using one-dimensional regression and machine learning for the calibration of the sensors. For the outdoor experimental data analysis, we used a dataset provided in a published work that conducted the experiment with several low-cost gas sensing platforms, from which we used a carbon dioxide NDIR sensor and the available climatic data.

It was found that the low-cost sensors, when used in a “ready-to-use” scenario, that is, uncalibrated by the end-user, may not meet their nominal accuracy parameters. When using one-dimensional calibration, such as linear regression or inverse power law, the accuracy was slightly enhanced, but we found that the size of the training subset used to extract the calibration equation did not lead to higher accuracy. Furthermore, it was observed that in the outdoor environment, where the sensors are exposed to more unstable conditions, linear regression is less accurate than indoors. Low-cost CO_2_ sensors with accurate readings outdoors can be a resourceful asset to the evaluation of human-caused environmental impacts in urban centres, such as CO_2_ accumulation in heat islands due to the lack of ventilation, or the incidence in residential blocks near roads or industries may take advantage of the ML-calibrated readings.

It was also observed that even a relatively small quantity of data (e.g., 20% of the dataset), but with significant data diversity, sufficed to provide useful information to the ExtRa-Trees ensemble regressor model to achieve satisfactory accuracy. This finding occurred in both indoor and outdoor experiments. However, the indoor environment is known to provide less variation in its environmental data than outdoors, and, for this reason, it is not recommended to use a calibration model that was trained with indoor sensor data in outdoor applications; the opposite situation is also true, considering that the variation in carbon dioxide is smaller outdoors than indoors and this can be ineffective to correct higher concentrations readings.

In the indoor experiment, although the MOS sensor was tricky to use and difficult to obtain information from its output using their nominal information, the ML model was able to effectively calibrate them and even compensated for the unexpected behaviour of one sensor (MOS sensor unit 1), extracting information from the very compressed and noisy sensor output. The NDIR sensors, in turn, were more user-friendly, since they had a built-in calibration feature to establish the base value of 400 ppm when exposed to fresh air and are available to the end-user with a ready-to-use inter-integrated circuit (I^2^C) interface, providing readings in parts per million. The sensors also showed no significant accuracy variation above or below the threshold of 1500 ppm (see Table 9), suggesting that the ML model is stable along the measurement range.

It is worth mentioning that low-cost sensors, whether using MOS or NDIR transduction principles, are instruments available to the end-user at affordable prices and with the premise of being efficient solutions for monitoring carbon dioxide when compared to reference instruments. Therefore, these sensors may be currently used in scenarios where the user is voluntarily involved in the development of the artefact with the simple intention of sharing his data or conducting his own research (for example, in participatory sensing, Internet of Things, and Makers Movement contexts). For this reason, likely, much of the available data on air quality using low-cost sensors were generated by sensors like those evaluated in this work. Given this, we have shown that low-cost sensors readings, when taken raw, can be very noisy and inaccurate, but even so, it is possible to achieve high accuracy using these cheaper devices resorting to features such as a beacon sensor (even if for a limited time) or a nearby reference station using a machine learning algorithm for calibration.

An important finding obtained from the outdoor experiment was that the total usage time of a sensor exposed to external conditions is a piece of valuable information that can contribute to optimising the error reduction using the algorithm. Also, we observed that at the same time, highly diverse training data are relevant for algorithm training; this can be quite challenging to obtain since it requires long-term experiments in real-world scenarios or controlled environments (e.g., climatic chambers) capable of providing larger environmental data combinations in shorter terms.

All analyses made in this paper were conducted in the Python language using the “sci-kit learn”, “sci-py”, “stats model”, and “matplotlib” packages. The advantage of using the machine learning models available in Python is that they can be embedded into portable devices running for real-time calibration. This feature can also be easily put into an online server application for a sensor network application.

Concerning the limitations of this work, we acknowledge that our sample of sensor units used in the indoor experiment, due to its reduced size, is not a robust representation of currently available low-cost CO_2_ sensors. A long-term indoor investigation using more sensors with more diverse situations (combinations of carbon dioxide, temperature, and relative humidity) is being considered as future work. Considering that the data we used for the outdoor experiment were obtained from a single sensor model, an outdoor investigation using sensors with different transduction principles from different manufacturers can also be considered. Regarding the monotonic drift investigation, as sensor ageing occurs slowly, the usage of a large and long-lasting continuous block of data would be a positive investigation to more deeply assess the influence of sensor exposure time on multidimensional sensor calibration.

## Figures and Tables

**Table 1:** Numerical information of sensor performance both in raw conditions and direct calibration.

Sensor Unit and Dataset		MAE (ppm)	RMSE (ppm)	r	ρ
MOS #1	Raw	N/A	N/A	−0.281	−0.186
	Calib.	727	1169	0.254	0.186
MOS #2	Raw	N/A	N/A	−0.891	−0.793
	Calib.	132	163	0.991	0.793
NDIR #1	Raw	67	94	0.999	0.981
	Calib.	45	60	0.999	0.981
NDIR #2	Raw	232	337	0.926	0.941
	Calib.	92	113	0.996	0.941

**Table 2:** Averaged performance of the machine learning algorithm validation (k = 5; training subset size = 20%).

Sensor Unit	MAE (ppm)	RMSE (ppm)	r	ρ
MOS #1	51	102	0.996	0.942
MOS #2	27	54	0.999	0.990
NDIR #1	21	39	0.999	0.991
NDIR #2	25	51	0.999	0.990

**Table 3:** Numerical performance of sensor accuracy in raw utilization and after linear regression.

Sensor Unit	MAE (ppm)		RMSE (ppm)		*r*		*ρ*		Total Exposition Time (Days)
	Raw	Lin.Reg.	Raw	Lin.Reg.	Raw	Lin.Reg.	Raw	Lin.Reg.	
#1	322	20	473	30	0.243	0.242	0.239	0.239	58.2
#2	88	24	123	33	0.295	0.295	0.332	0.332	55.9
#3	248	11	370	18	0.230	0.231	0.356	0.356	24.8
#4	79	21	149	30	0.240	0.240	0.277	0.277	114.1
#5	46	23	61	32	0.544	0.544	0.361	0.361	47.0
#6	242	21	349	31	0.294	0.294	0.272	0.272	76.3
#7	41	19	49	28	0.525	0.525	0.332	0.332	65.5
#8	493	22	558	28	0.160	0.160	0.185	0.185	26.1
#9	241	25	315	34	0.216	0.216	0.235	0.235	52.7
#10	465	23	567	29	0.245	0.245	0.211	0.211	48.8
#11	289	24	427	33	0.151	0.151	0.221	0.221	60.0
#12	133	16	201	25	0.258	0.258	0.224	0.224	80.3
#13	35	17	50	25	0.314	0.314	0.143	0.143	78.2
#14	192	19	236	25	0.259	0.259	0.270	0.270	40.6

**Table 4:** Numerical performance of sensor accuracy after the use of ExtRa-Trees.

Sensor Unit	MAE (ppm)	RMSE (ppm)	r	*ρ*
#1	6	12	0.922	0.868
#2	6	10	0.957	0.933
#3	3	6	0.951	0.883
#4	7	12	0.930	0.895
#5	7	11	0.955	0.924
#6	7	13	0.920	0.877
#7	5	9	0.960	0.898
#8	7	12	0.913	0.917
#9	7	12	0.937	0.921
#10	9	15	0.882	0.867
#11	6	11	0.948	0.914
#12	6	10	0.921	0.886
#13	5	9	0.943	0.901
#14	6	11	0.911	0.915

**Table 5:** Changes in the algorithm’s performance metrics after the removal of sensor exposition time from the input parameters.

Sensor Unit	MAE Increase	RMSE Increase	Change in *r* (From → to)
#1	100%	75%	0.922 → 0.720
#2	67%	50%	0.957 → 0.897
#3	100%	67%	0.951 → 0.846
#4	71%	58%	0.930 → 0.790
#5	57%	73%	0.955 → 0.866
#6	71%	62%	0.920 → 0.753
#7	100%	78%	0.960 → 0.867
#8	71%	42%	0.913 → 0.793
#9	57%	42%	0.937 → 0.871
#10	44%	33%	0.882 → 0.748
#11	67%	45%	0.948 → 0.875
#12	33%	40%	0.921 → 0.841
#13	60%	56%	0.943 → 0.844
#14	67%	55%	0.911 → 0.779

**Table 6:** Comparison of the sensor exposition time’s influence on the algorithm’s accuracy performance through the sensor relative measurement error analysis. Results from linear regression calibration are included.

Sensor Unit	Confidence Interval (95%) of the Sensor Relative Measurement Error		
	ML with Exposition Time	ML without Exposition Time	Linear Regression
#1	±4.6%	±8.7%	±11.5%
#2	±4.4%	±6.6%	±13.1%
#3	±2.4%	±4.4%	±7.5%
#4	±5.1%	±8.0%	±12.0%
#5	±4.6%	±7.8%	±12.8%
#6	±5.2%	±8.8%	±12.0%
#7	±3.9%	±6.8%	±11.1%
#8	±4.6%	±7.1%	±11.7%
#9	±4.8%	±6.9%	±13.3%
#10	±5.8%	±8.1%	±11.9%
#11	±4.2%	±6.6%	±12.8%
#12	±3.9%	±5.7%	±10.0%
#13	±3.6%	±5.9%	±10.1%
#14	±4.6%	±7.1%	±10.6%

**Table 7:** Indoor environmental conditions (*µ* = average; *σ* = standard deviation) matching the periods annotated in Figure 26, and the corresponding sensor conversion curve coefficients.

Interval (Hours)	Temperature (°C)		Humidity (%)		Conversion Curve. [F(x) = A × x^B^]	
	*µ_t_*	*σ_t_*	*µ_h_*	*σ_h_*	A	B
0–15	22.6	0.81	40.6	1.7	2.5 × 10^−3^	−7.5
15–45	21.4	0.89	39.4	1.7	4.5 × 10^−6^	−11.8
50–60	24.1	0.51	54.2	4.0	1.2 × 10^−6^	−14.1
70–90	24.8	0.56	48.3	3.0	1.0 × 10^−3^	−9.2
90–110	23.2	0.96	27.3	2.2	4.31	−3.4
110–135	22.8	0.88	21.8	1.6	1.0 × 10^−16^	−30.6

**Table 8:** Error reduction on accuracy parameters obtained by the ExtRa-Trees model in comparison to the one-dimensional calibration in the indoor experiment.

Accuracy Parameter	Sensor Unit			
	MOS #1	MOS #2	NDIR #1	NDIR #2
MAE	93%	79%	53%	73%
RMSE	91%	66%	33%	54%

**Table 9:** **The** 95% confidence interval of the relative sensor measurement error within the example protection limits criteria.

Sensor	<1500 ppm		>1500 ppm	
	ExtRa-Trees	Regression	ExtRa-Trees	Regression
MOS #1	±24.3%	±140% ^1^	±12.6%	±35.0%
MOS #2	±8.5%	±46.3%	±7.5%	±11.3%
NDIR #1	±6.4%	±15.0%	±5.1%	±6.2%
NDIR #2	±7.2%	±33.6%	±7.3%	±9.3%

^1^—This sensor unit showed a high bias after curve-fit calibration. Its relative measurement error interval was contained between −0.6 and +2.22.

**Table 10:** Relative error mitigation obtained via both calibration methods used in comparison to the raw error of sensors’ units against the reference.

Sensor Unit	MAE Reduction		RMSE Reduction	
	Lin.Reg	ExtRa-Trees	Lin.Reg	ExtRa-Trees
#1	93.8%	98.1%	93.7%	97.5%
#2	72.7%	93.2%	73.2%	91.9%
#3	95.6%	98.8%	95.1%	98.4%
#4	73.4%	91.1%	79.9%	91.9%
#5	50.0%	84.8%	47.5%	82.0%
#6	91.3%	97.1%	91.1%	96.3%
#7	53.7%	87.8%	42.9%	81.6%
#8	95.5%	98.6%	95.0%	97.8%
#9	89.6%	97.1%	89.2%	96.2%
#10	95.1%	98.1%	94.9%	97.4%
#11	91.7%	97.9%	92.3%	97.4%
#12	88.0%	95.5%	87.6%	95.0%
#13	51.4%	85.7%	50.0%	82.0%
#14	90.1%	96.9%	89.4%	95.3%

## Data Availability

The proprietary dataset used in this manuscript is available in Zenodo Repository, under the DOI 10.5281/zenodo.8111111. The third-party dataset used in this manuscript is available in Zenodo Repository, under the DOI 10.5281/zenodo.1146109.
